# Novel Endoscopic Suture Anchor Device for Large Defect Closure Following Endoscopic Submucosal Dissection

**DOI:** 10.5152/tjg.2025.25240

**Published:** 2025-10-31

**Authors:** Jiancong Feng, Yaqi Zhai, Zhenyu Liu, Enqiang Linghu

**Affiliations:** 1 Department of Gastroenterology, The First Medical Center of Chinese PLA General Hospital, Beijing, China

## Case Presentation

**Figure 1.**
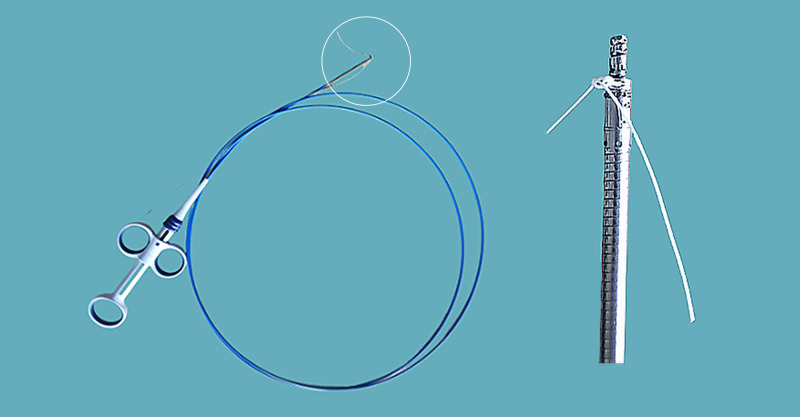
A novel endoscopic suture anchor device was engineered.

**Figure 2.**
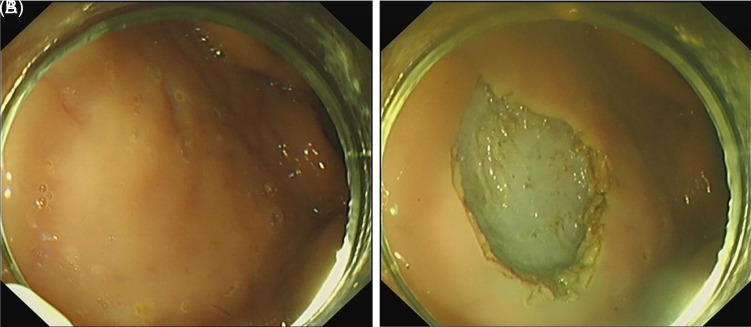
A mucosal defect following simulated endoscopic submucosal dissection.

Endoscopic submucosal dissection (ESD) allows for en bloc resection of gastrointestinal polyps and early cancers. Large post-ESD defects require meticulous management to prevent complications, including delayed bleeding and perforation. Endoscopic closure represents a promising strategy for mitigating these adverse events.^1^ However, closure of large post-ESD mucosal defects remains a significant clinical challenge. Although endoscopic suturing devices have advanced, their widespread adoption is limited by technical complexity, regional disparities in accessibility, and suboptimal cost-effectiveness.^2^^,^^3^ Through integrated analysis of the clinical challenge with corkscrew mechanical principles, a novel endoscopic suture anchor device was engineered (Figure 1). Its technical feasibility underwent preliminary ex vivo assessment using fresh porcine gastric specimens sourced from a certified abattoir. Following simulated ESD, a mucosal defect measuring 3 × 2 cm was created at the anterior wall of the gastric body (Figure 2) and closed it using the novel suture anchor device. This study utilized exclusively ex vivo porcine stomachs without live animal involvement, fully complying with the Replacement principle; thus, Animal Ethics Committee approval was unnecessary. Ethical procurement from commercial sources precluded consent requirements per institutional guidelines.

## Technique

**Figure 3.**
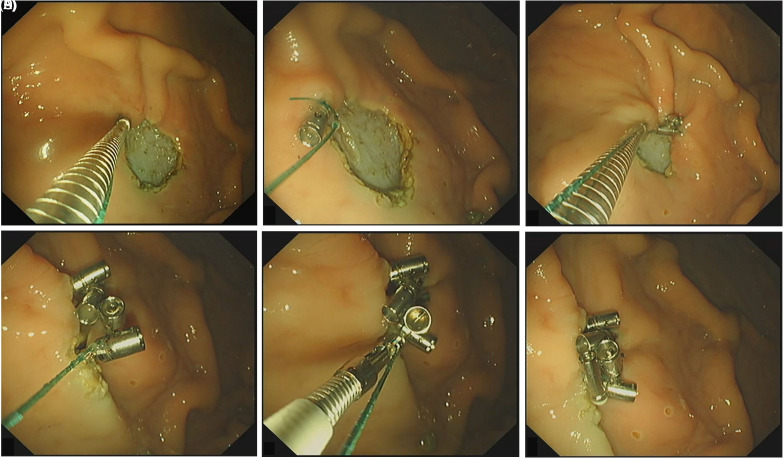
The steps for defect closure using the novel endoscopic suture anchor. a: The first anchor was fixed at the wound edge mucosa and screwed into the tissue; b: The anchor was released by pressing the handle; c: Subsequent anchors were advanced and released in the same way; d: Applying proper tension to optimize tissue apposition; e: The cinching device approximated the defect edges, then the suture was cut; f: Complete defect closure was achieved.

The novel suture anchor device (anchor diameter: 1.6 mm; thread pitch: 1.0 mm) was fabricated by Micro-Tech (Nanjing, China). It can be directly delivered through a 3.2 mm endoscope working channel and deployed using a GIF-Q260J endoscope (Olympus, Tokyo, Japan), and can be combined with sutures to achieve defect closure according to the following steps (Figure 3, Video 1). First, a suture anchor pre-threaded with polymerized nylon suture was introduced through the endoscopic channel and positioned at the mucosal defect margin. The handle was rotated clockwise to embed the anchor securely within the tissue. Gentle traction confirmed stable fixation prior to anchor release via handle depression. Subsequently, a second suture-loaded anchor was advanced through the channel and similarly implanted into the contralateral mucosal margin. Following this deployment, suture tension was meticulously optimized. The procedure was replicated for the placement of three additional anchors. After achieving appropriate tension to facilitate optimal tissue apposition without suture compromise, a cinching device was advanced along the suture to ensure complete defect edge approximation, followed by suture transection. Ultimately, successful defect closure, characterized by the absence of submucosal gaps, was attained.

## Conclusion

This ex vivo experiment demonstrates the feasibility of the endoscopic suture anchor device in achieving complete closure of a large post-ESD defect. Distinct from the X-Tack system’s platform-dependent delivery of four preloaded helical suturing tacks,^4^ this novel device offers on-demand individual anchor deployment, providing clinical flexibility for both interrupted and continuous suture patterns. Furthermore, leveraging a conventional through-the-scope clip platform, this novel suture anchor device demonstrates superior potential cost-benefit profiles. While these preclinical results highlight the device’s technical feasibility and procedural flexibility, subsequent validation through in vivo survival studies, which are currently being conducted, and comparative clinical trials remains essential to evaluate its efficacy and safety relative to conventional closure approaches.
